# Applying MALDI-TOF MS to resolve morphologic and genetic similarities between two *Dermacentor* tick species of public health importance

**DOI:** 10.1038/s41598-024-69768-8

**Published:** 2024-08-27

**Authors:** Maria F. B. M. Galletti, Joy A. Hecht, John R. McQuiston, Jarrett Gartin, Jake Cochran, Bessie H. Blocher, Bryan N. Ayres, Michelle E. J. Allerdice, Lorenza Beati, William L. Nicholson, Alyssa N. Snellgrove, Christopher D. Paddock, US Tick MALDI-TOF consortium, Ashley Kennedy, Goudarz Molaei, Paula Lado, Janet Foley, Jerome Goddard, James L. Occi, Kerry Padgett, Elizabeth Dykstra, Melissa Nolan, Roberto Cortinas, Samantha Sambado, Sarah Fink, Scott R. Campbell, Yamila Romer

**Affiliations:** 1 Rickettsial Zoonoses Branch, Division of Vector-Borne Diseases, National Center for Emerging and Zoonotic Infectious Diseases, Centers for Disease Control and Prevention, Atlanta, USA; 2 Division of High-Consequence Pathogens and Pathology, National Center for Emerging and Zoonotic Infectious Diseases, Centers for Disease Control and Prevention, Atlanta, USA; 3 United States National Tick Collection, Institute for Coastal Plain Science, Georgia Southern University, Statesboro, USA; 4 Delaware Mosquito Control Section, Newark, USA; 5 Connecticut Agricultural Experiment Station, New Haven, USA; 6 The Ohio State University, Columbus, USA; 7 University of California-Davis, Davis, USA; 8 Mississippi State University, Mississippi State, USA; 9 Rutgers University, New Brunswick, USA; 10 California Department of Public Health, Richmond, USA; 11 Washington State Department of Health, Olympia, USA; 12 University of South Carolina, Columbia, USA; 13 University of Nebraska-Lincoln, Lincoln, USA; 14 University of California Santa Barbara, Santa Barbara, USA; 15 Franklin County Public Health Columbus, Columbus, USA; 16 Suffolk County Department of Health Services, Yaphank, USA; 17 Emory University, Atlanta, USA

**Keywords:** MALDI-TOF, Reference database, Tick, *Dermacentor*, Public health, Mass spectrometry, Entomology

## Abstract

Hard ticks (Acari: Ixodidae) have been historically identified by morphological methods which require highly specialized expertise and more recently by DNA-based molecular assays that involve high costs. Although both approaches provide complementary data for tick identification, each method has limitations which restrict their use on large-scale settings such as regional or national tick surveillance programs. To overcome those obstacles, the matrix-assisted laser desorption/ionization time-of-flight mass spectrometry (MALDI-TOF MS) has been introduced as a cost-efficient method for the identification of various organisms, as it balances performance, speed, and high data output. Here we describe the use of this technology to validate the distinction of two closely related *Dermacentor* tick species based on the development of the first nationwide MALDI-TOF MS reference database described to date. The dataset obtained from this protein-based approach confirms that tick specimens collected from United States regions west of the Rocky Mountains and identified previously as *Dermacentor variabilis* are the recently described species, *Dermacentor similis*. Therefore, we propose that this integrative taxonomic tool can facilitate vector and vector-borne pathogen surveillance programs in the United States and elsewhere.

## Introduction

Hard ticks (Acari: Ixodidae) transmit the most diverse collection of arthropod-borne viruses, bacteria, and protozoan pathogens on earth, and are second only to mosquitoes as arthropods of greatest medical and veterinary importance^1,2^. As of 2023, 26 etiologically and epidemiologically distinct tickborne diseases of humans have been identified in the United States; remarkably, 11 of these diseases have been discovered since 2000^3–6^. Coupled with these discoveries has been a reexamination of several hard tick species in the United States that, until relatively recently, were considered largely irrelevant or noncontributory as vectors of agents pathogenic to humans^7^. Moreover, the taxonomic methods that most accurately define a particular tick species have been reevaluated^8^. Historically, tick taxonomy relied almost exclusively on morphological and morphometric characteristics to separate species. This approach, identified as the typological species concept, forms the basis for formal scientific descriptions of species and the development of local and regional dichotomous keys^8^. Despite the routine use of those keys by non-taxonomists and public health communities worldwide^8^, challenges may surge while identifying novel or invasive tick species^9,10^. A more nuanced approach, embraced increasingly by tick taxonomists on multiple continents, combines various morphological, genetic, ecological, behavioral, and geographical differences to define a particular species^11–14^. The application of integrative taxonomy to ticks of medical and veterinary importance has revealed greater species diversity than appreciated by the conventional typological approach and, in some cases, has provided information that better describes the distribution and environmental determinants of tickborne diseases^12^. However, distinct challenges exist in the construction of complex data sources combined most frequently with the integrative approach. These include highly specialized expertise and training required of tick taxonomists to identify subtle morphological features often associated with closely related species^8,13^, as well as costly procedures needed to generate large sets of genetic targets or complete tick genomes to provide enough genetic granularity to accurately separate these closely related, yet ecologically and epidemiologically distinct species of hard ticks^13,15,16^.

The American dog tick, *Dermacentor variabilis* (Say, 1821), is among the most widely distributed, three-host hard tick species in North America. An avid human biter, it is the third most frequently identified tick infesting humans in the United States^17^ and is a vector of multiple zoonotic pathogens, including *Rickettsia rickettsii* (the agent of Rocky Mountain spotted fever), *Francisella tularensis* (the agent of tularemia), and an *Anaplasma bovis*-like species^6,18,19^. Also, the species is associated with tick paralysis in humans and other animals^1,20^. For more than a century, investigators strictly applied the typological species concept to identify specimens of *D. variabilis*, thereby indicating a geographical distribution for this species that spanned the continental United States^21–26^. However, as early as 1910, Wardell Stiles described considerable variation in respect to spiracular plate features in the genus *Dermacentor*, being the first suggestion on the use of those variations as a basis for describing new species or subspecies^27^. More recently, investigators gradually recognized that distinctive physiological and ecological characters differentiated populations of *D. variabilis* west of the Rocky Mountains from those in the eastern United States and suggested that these two populations could be separate taxa^28–31^. These observations were recently confirmed by an integrative taxonomic approach that combined morphological and high-throughput DNA-based identification methods to formally separate the western clade as *Dermacentor similis* from the eastern clade, *D. variabilis*^32^.

During the last decade, investigators have increasingly applied matrix-assisted laser desorption/ionization time-of-flight mass spectrometry (MALDI-TOF MS) as an additional integrative tool for the identification of certain hard tick species from Europe, Africa, and Asia^33–38^. This technique analyzes large biomolecules to create protein spectra that are unique to a particular species and life stage of the tick. In this context, MALDI-TOF MS can provide accurate data from specimens traditionally challenging to morphological or nucleic acid-based evaluations to include immature stages, damaged, or archival specimens stored in ethanol for several years^33,39,40^. Despite its accuracy, precision, specificity, sensitivity, and low-cost, MALDI-TOF MS has been used infrequently as a taxonomic tool for hard ticks of the Western Hemisphere^41^. The absence of a widely available, suitable, and broad reference database is a limiting factor^42^. Also, to our knowledge, there have been no validated applications of this technique to medically relevant tick species in the United States. Herein, we harnessed MALDI-TOF MS to rapidly, inexpensively, and accurately differentiate *D. variabilis* from *D. similis*. We further created a national and publicly accessible database for these species. Continued development of this reference database and implementation of the technology in public health laboratories could leverage a rapid, low cost, and widely available tick identification technique that enhances and supports national efforts to provide accurate and predictive tick and tickborne pathogen surveillance^7^.

## Results

### Morphological identification of *Dermacentor* spp.

**Figure 1:**
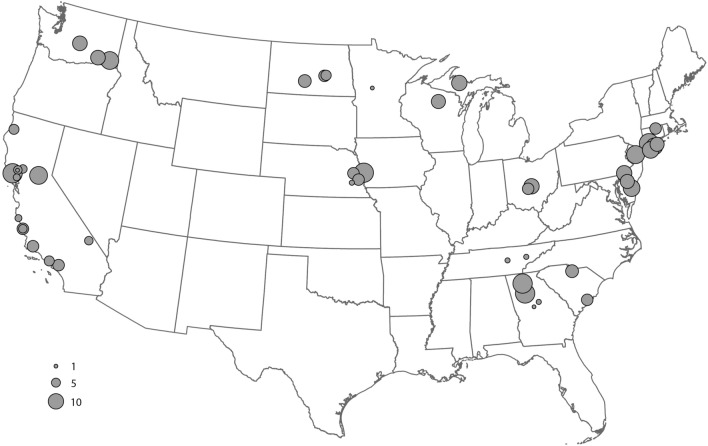
Distribution map of collection sites of *Dermacentor variabilis* and *Dermacentor similis* adult specimens evaluated by MALDI-TOF MS. Number of specimens per site is indicated by circle size.

**Figure 2:**
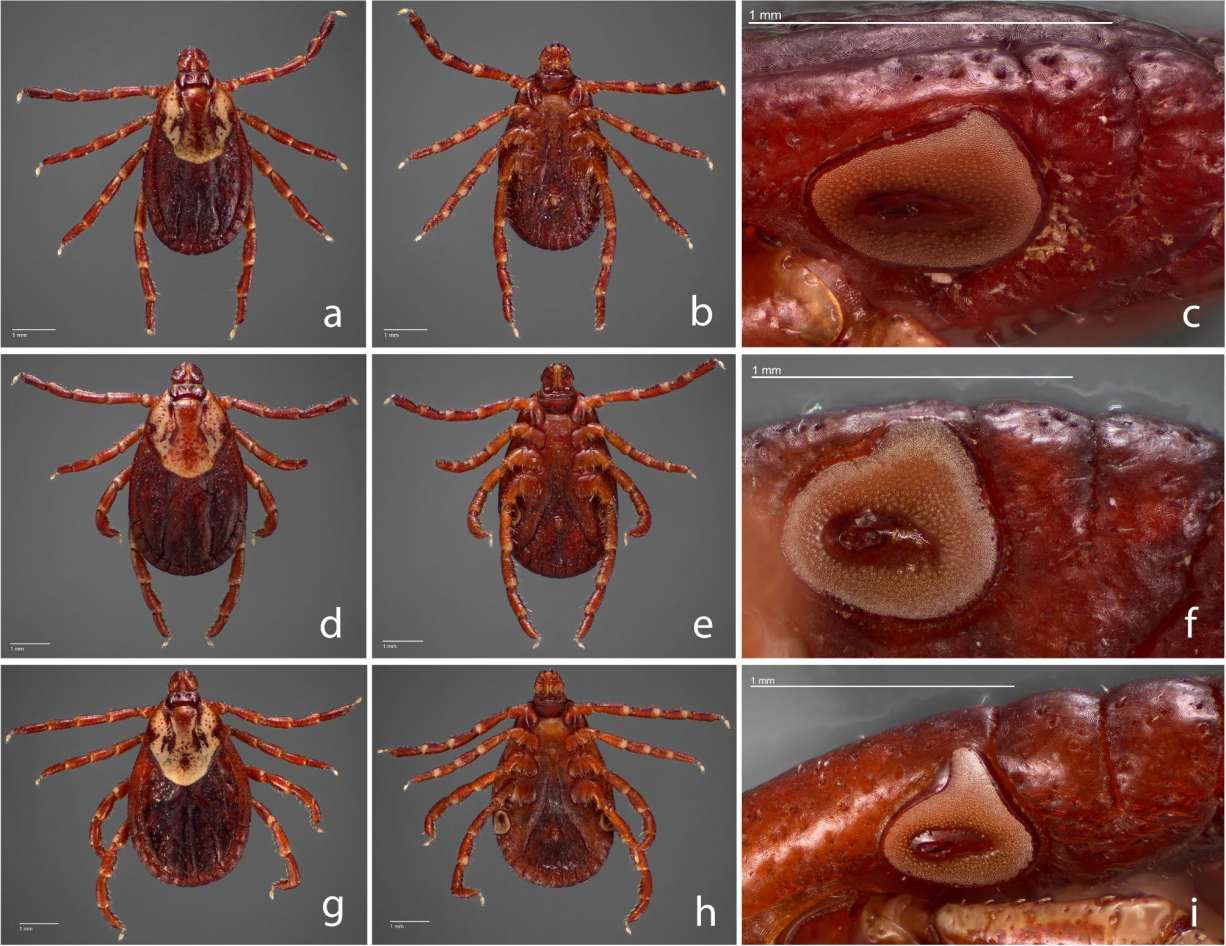
*Dermacentor* female images. *Dermacentor variabilis* female dorsal (**a**) and ventral (**b**) from New York, female dorsal (**d**) and ventral (**e**) from North Dakota, and *Dermacentor similis* female (**g**) dorsal and ventral (**h**) from California. (**c**), (**f**), and (**i**) represent the spiracular plate of the respective specimen to the left.

We evaluated 492 adult *Dermacentor* spp. ticks, comprising 249 females and 243 males obtained from 19 US states (Fig. 1), to create the morphological, gene-based, and protein-based datasets for this study. Species identity was determined for each specimen by trained medical entomologists using morphological characteristics described in established taxonomic keys^43,44^. The morphological analysis identified consistent morphological differences between specimens collected in the western US and those from the eastern and midwestern US (Fig. 2), as described in previous studies^32,43^.

Additional corroborative evidence for a *Dermacentor* species in the western United States morphologically distinct from *D. variabilis* was acquired by a separate evaluation of 207 adult specimens (123 females and 84 males) archived in the United States National Tick Collection. Those specimens originally identified as “*Dermacentor variabilis*” were collected between 1910 and 1986 from California (n = 46), Idaho (n = 36), Oregon (n = 31), and Washington (n = 94). Although no molecular-based work was performed with this group, the morphology of each of these specimens corresponded to the characteristics described for *D. similis*, and none were identified as *D. variabilis*^32^ (Fig. S1).

### DNA-based phylogenetic analyses

**Figure 3:**
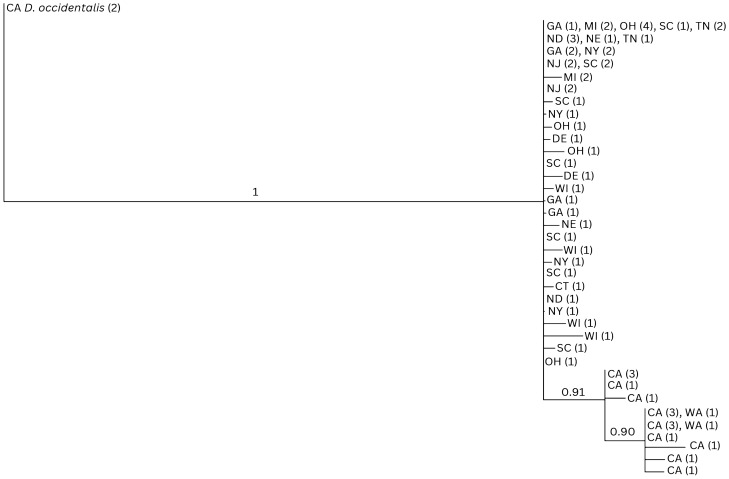
Bayesian analysis (BA) phylogenetic tree of concatenated ITS2 nuclear DNA and 12S ribosomal DNA (631 bp) from 66 sequenced specimens. The top clade consists of the *Dermacentor variabilis* from states across the Midwest and Eastern US; the bottom clade represents western *Dermacentor similis* samples from Washington (WA) and California (CA). Numbers on nodes represent posterior probabilities. Numbers in parentheses represent the number of identical sequences within that particular US state. *Dermacentor occidentalis* (Do) specimens from California were used as an outgroup. CT: Connecticut; DE: Delaware; GA: Georgia; MI: Michigan; ND: North Dakota; NE: Nebraska; NJ: New Jersey; NY: New York; OH: Ohio; SC: South Carolina; TN: Tennessee; WI: Wisconsin.

A subset of 105 specimens from 12 states identified by morphological criteria were evaluated by DNA sequencing of segments of the nuclear ITS2 and mitochondrial 12S ribosomal DNA genes. A total of 344 base pairs of the ITS2 and 287 base pairs of the 12S rDNA genes were successfully sequenced from 86 and 73 samples, respectively. Of these, a total of 66 samples were successfully sequenced for both targets and are represented in Figs. 3 and S2.

The phylogenetic reconstructions based on the ITS2 nuclear DNA and 12S ribosomal DNA segments failed to separate the two species. Indeed, in both cases, specimens belonging to *D. similis* and *D. variabilis* clustered within the same basal polytomy (Figs. 3 and S2). The analysis of the concatenated dataset (66 ticks) provided a better resolution. While the *D. variabilis* lineages formed a large polytomy with no indication of them belonging to a monophyletic cluster, all *D. similis* lineages grouped in a single supported (91% posterior probability) clade originating within the *D. variabilis* polytomy. Although the concatenated dataset indicates that the California *D. similis* samples may constitute a separate lineage, the fact that the two species do not segregate into separate monophyletic mutually exclusive clades does not allow for them to be considered separate taxa, based solely on the recognized phylogenetic species concept.

In ITS2, the *D. similis* samples all exhibit three single nucleotide polymorphisms relative to the *D. variabilis* samples, including one transition from adenine to guanine and two transitions from guanine to adenine (Table S2). The Washington samples are all identical to each other, as are most of the California samples, although four *D. similis* (one specimen from Sonoma County and three specimens from Lake County in California) each have single polymorphisms that are unique among the *D. similis* samples.

Sequences for 12S exhibit more variation within both *D. similis* and *D. variabilis*; however, consistent polymorphisms separating the two species are clear. All samples of *D. similis* exhibit an adenine to thymine transversion and a thymine to cytosine transition that are not present in *D. variabilis*. Additionally, except for two specimens from California, all *D. similis* exhibit a second thymine to cytosine transition relative to *D. variabilis*, described in Table S2.

### Protein reference database construction using MALDI-TOF MS analysis data

**Figure 4:**
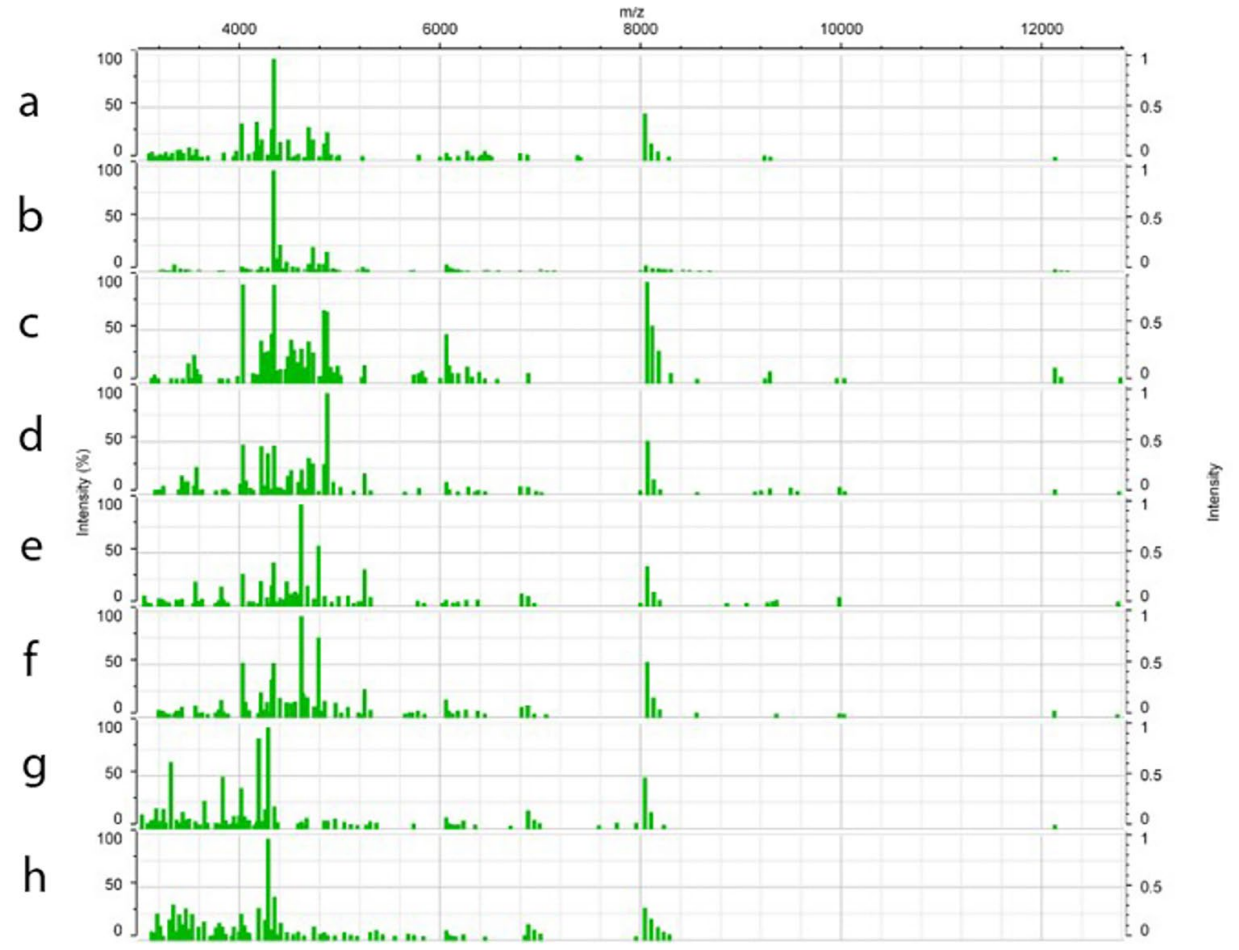
Representative MALDI-TOF mass spectra over the entire mass range of *Dermacentor variabilis* female and male specimens from New York (**a**,**b**), a female and male *D. variabilis* from North Dakota (**c**,**d**), a female and male *Dermacentor similis* from California (**e**,**f**), and as an outgroup a female and male *Dermacentor occidentalis* from California (**g**,**h**). The x-axis represents mass/charge ratios (m/z) and the y-axis arbitrary units of peak intensity.

A set of four legs per specimen, dissected from 402 morphologically identified *D. variabilis* and *D. similis*, were individually analyzed using MALDI-TOF MS. Following quality control parameters for database construction, single mass spectra from 54 samples (13.43%) were discarded. For each specimen added to the reference database, a minimum of 20 single mass spectra comprised the main spectra profile (MSP). Additionally, all specimens integrated in the reference database were screened by real-time PCR assays for pathogens/endosymbionts described in Table S1, and only negative samples were included in the database to eliminate any probable interference of bacterial protein in the tick protein profiles for identification purposes. A total of 7.75% (27/348) of the analyzed samples were positive and therefore discarded from any further analysis. Figure 4 shows the main spectra profiles obtained from representative specimens in the database. High-quality spectra from 321 ticks, comprising specimens obtained from 15 states collected from the historically accepted range of *D. variabilis* in the United States, were selected to construct the database.

**Figure 5:**
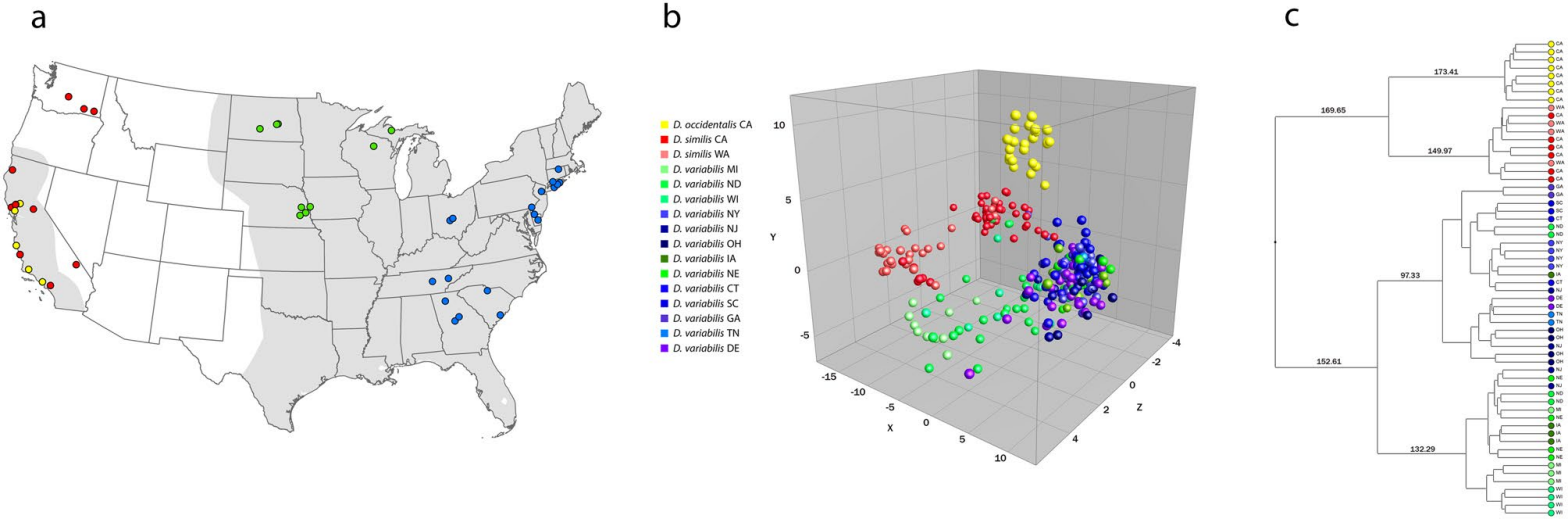
MALDI-TOF MS reference database analysis. Blue and purple variants represent *Dermacentor variabilis* from eastern US, green variants *D. variabilis* from midwestern US, red variants *D. similis* from California and Washington, and yellow represent *D. occidentalis* from California used as an outlier. (**a**) Geographical distribution of samples integrated in the reference database. Estimated historical distribution of *D. variabilis* is highlighted in gray. (**b**) Spectrum-based principal component analysis (PCA) from all spectra present on the *Dermacentor* reference database generated on Bionumerics 7.6. The contributions of X, Y and Z were 29.9%, 12.0% and 7.2%, respectively. (**c**) MSP dendrogram of 60 database selected specimens; numbers are distance units to the relative similarity of the MS spectra. Color code for (**b**) and (**c**) are described in the PCA legend.

With a representational database constructed, the main spectra profiles comprising the database were then evaluated to determine if intergroup differentiation was possible. Highly similar MSP were identified during visual examination, allowing for differentiation of the specimens from the western US as well as between the midwestern and eastern regions (Fig. 4). In addition, specimens belonging to the same region shared several mass peaks. This grouping was first confirmed by Principal Component Analysis (PCA), which identified interspecies specificity between 73 specimens from the western US as well as among the 223 specimens from the midwestern and eastern regions (Fig. 5A). This was also confirmed by the relative similarity highlighted in the PCA and dendrogram (Fig. 5B,C).

Although many of the major species-specific peaks were also highly conserved within a given cluster, supporting the grouping (Fig. 5), heterogenicity between the midwestern (green variants) and eastern (blue and purple variants) populations was identified and allowed for intra-group differentiation. Main spectra profile analysis did not allow for differentiation between sexes within these groups, a finding that was also seen in previously published data^39^. The 321 protein main spectra profiles generated for the construction of the MALDI-TOF MS reference database have been deposited and are publicly available for search at CDC MicrobeNet tools (MicrobeNet).

### MALDI-TOF MS tick identification and database validation

**Figure 6:**
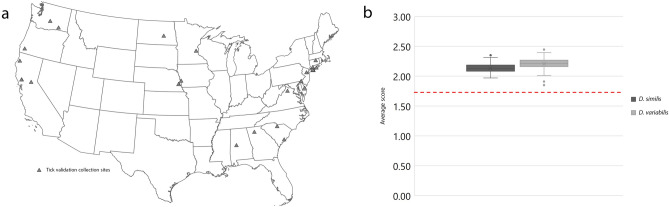
*Dermacentor* specimens used for the MALDI-TOF reference database validation. (**a**) Geographical distribution of specimens identified in the study. Each gray triangle represents a collection site. (**b**) Distribution of the log score values obtained from the real time classification of 26 *Dermacentor similis* and 64 *Dermacentor variabilis* against the reference database of MALDI-TOF main spectra profiles*.* All specimens score > 2.0 except three *D. similis* between 1.97 and 1.98 and two *D. variabilis* between 1.85 and 1.91. Red dashed line represents the minimum score log for high confidence species level identification.

Spectra of an additional 90 samples were acquired for identification and database validation. Within those samples, 26 originated from western locations and 64 from midwestern and eastern states, representing a total of 15 states (Fig. 6A). In order to evaluate if changes in sample characteristics would influence the identification process, 86.66% of tested specimens followed all the standardized characteristics adopted for the database construction, and the remaining samples used were stored at different conditions including (i) two older samples stored for 7 years in 70% ethanol at room temperature, (ii) eight samples kept at refrigerated (4–10 °C) temperature for 4 months until processing, and (iii) two partially engorged specimens. All tested samples generated excellent quality spectra, which were queried against the reference MSP database described above. The validation revealed that 100% of the *D. variabilis* and *D. similis* were correctly identified and fully agreed with the morphological data initially acquired. The identified MALDI-TOF MS log score values (LSV) averaged 2.21 ± 0.107 for *D. variabilis* and 2.14 ± 0.096 for *D. similis* (Fig. 6B). No difference was identified in LSV between samples with different storage conditions and the standardized group kept at the same conditions as the original reference database.

### Pathogen screening of the MALDI-TOF MS validation specimens

Each of the 90 samples used for the MALDI-TOF MS reference database validations was screened for Rickettsiaceae and Anaplasmataceae species. Six (6.66%) samples revealed an agent from one of these bacterial families, including two *D. similis* from California infected with *Rickettsia bellii*, one *D. variabilis* from South Carolina infected with *Rickettsia parkeri*, one *D. variabilis* from New York infected with *R. montanensis*, and two *D. variabilis* specimens from Iowa positive for an *Anaplasma bovis*-like agent^6^. This additional analysis revealed that the specimen infection status did not impact the LSV or the overall identification.

## Discussion

MALDI-TOF MS has been used since the early 2000s to identify and speciate several micro-organisms, including bacteria and fungi that previously required biochemical or molecular assays for characterization and phylogenetic classification^45^. The broad acceptance of this method in medical microbiology is attributed to its simplified sample preparation method, fast and reliable data output, and competitive cost per sample^45^ when compared with conventional phenotypic and molecular methods. Previous study on the MALDI-TOF MS cost–benefit applied on routine microbiology detailed an annual net savings of 87.8% if compared to traditional biochemical-based methods, considering direct, indirect, and total costs^46^. The breakdown in reagents cost per sample in that study was $0.43 using MALDI-TOF compared to $3.59 for the traditional biochemical-based method. The caveat still relies on the instrument availability. Although its capital cost is still high for educational and research-only facilities, accessibility to shared and service-based laboratories may facilitate its use. On the other hand, its presence in medical facilities (e.g. hospitals) associated to reduced total costs has allowed the method to be established as a reference in diagnostic laboratories for the identification of microorganisms and it has revolutionized microbial identification^46–48^. Within the last several years, MALDI-TOF MS has been adapted to the identification of several groups of relevant arthropods^49,50^, including hard tick species^35–41,51^.

In this investigation, we used MALDI-TOF MS technology to examine two tick species medically relevant in North America that until recently had been characterized as a single species. Results of this work revealed the value of MALDI-TOF MS as an integrative taxonomic tool to support the recent separation of *D. similis*. from *D. variabilis*^32^. By creating a validated and robust dataset that included corresponding morphological and genetic information, we provide a proof-of-concept for this highly specific technique that leverages species identification of two medically relevant ticks. This is particularly notable for species that pose morphological or molecular-based challenges for rapid and accurate identification^12,14^. However, to precisely identify species, a well-curated and reliable reference specimen collection and molecular databases must be available^42^. To date, in-house MALDI-TOF tick protein databases have been constructed using a restricted number of specimens at research laboratories^42^. Although accessible through collaboration, the level of diversity and representation of those tick databases is limited, which could jeopardize a successful identification by not accounting for non-clonal organism diversity.

To overcome those obstacles, we constructed the first US nationwide MALDI-TOF protein-based reference database as a free online tool to allow broad accessibility. For high-quality data acquisition, strict sample features were adopted based on previous MALDI-TOF assessments as well as common practices used in the tick field for preservation and transportation of specimens^34,52^. Although 13.43% of generated MSP did not meet the intrinsic level of quality defined by the manufacturer for the construction of the reference database, we believe the loss wasn’t due to sample conditions as other specimens from the same lot were successfully integrated in the reference database. In addition to those conditions, the specimen non-infectious status was featured to prioritize database consistency. Lastly and most importantly, the group of selected specimens incorporated into the reference database was the most geographically representative of the historical *Dermacentor variabilis* distribution, to account for any differences present on those populations. Under these criteria, the analysis of the protein main spectra profile dataset created for each *D. variabilis* and *D. similis* species concurred with established morphological and DNA-based criteria. Indeed, the robustness of the reference database was validated by the accurate identification of specimens kept in different conditions and infected by different organisms. Additional studies on the influence of other tick-borne pathogens and storage conditions in addition to other tick species are needed to validate the implementation of the method on a large-scale surveillance program.

Based on the national reference database, the taxonomic resolution provided by MALDI-TOF was equivalent to, and perhaps greater than, conventional morphological and genetic techniques^53,54^ applied to the separation of these two species. In this context, MALDI-TOF also identified a probable transitional midwestern *D. variabilis* population not previously documented by morphology or conventional DNA-based methods, suggesting the enhanced granularity of this high-throughput protein-based technique. Interestingly, intra-group variation of *D. variabilis* in Wisconsin and Michigan was previously described using ddRAD-Seq^32^, which reinforces the strength of high-throughput methods for intra-group differentiation. As far as targeted DNA sequencing is considered, the analysis of fragments of ITS2 nuclear DNA and 12S ribosomal DNA failed to clearly separate the two species in the present study. Attempts to amplify additional gene targets such as 16S mitochondrial DNA were unsuccessful due to inconsistencies within target amplification among different *D. variabilis* populations. Therefore, we pursued the use of ITS2 nuclear DNA and 12S ribosomal DNA, each representing a different source of information (nuclear and mitochondrial), also important tools in the context of both taxonomic and phylogenetic studies. In regards to ITS2 nuclear DNA, sequencing a shorter fragment might have compromised the analysis although it has been successfully used for tick speciation previously^55^. Also, the difficulty in differentiating the species on a gene level may be a result of the very recent speciation event between *D. variabilis* and *D. similis*, with not enough mutagenic events to reflect the divergence, also observed in other species of recent speciation in the United States^13,56^. Nevertheless, phylogenetic analyses based on three mitochondrial gene sequences (12S rDNA, 16S rDNA, and COI) correctly identified two strongly supported, mutually exclusive clades for the two taxa and the discrepancy with the present study is possibly due to the wider taxonomic representation^31^.

Despite almost two decades of investigations that effectively applied MALDI-TOF MS to speciate ticks of medical and veterinary importance, even the most recent applications of this technology have not demonstrated its ability to specifically detect pathogenic agents associated with field tick specimens and instead relied on molecular methods to accomplish these crucial associations^5,40,57–59^. This is considered a remaining challenge for MALDI-TOF technology and is possibly attributed to the discrepancy between the low amount of bacterial protein compared to the high background of tick proteins in the tested sample. In contrast to purified and highly concentrated bacterial colonies evaluated by MALDI-TOF in diagnostic microbiology, bacterial proteins extracted from ticks represent a small fraction of the total specimen comprising mostly of tick proteins. Indeed, MALDI-TOF MS has been shown to identify pathogens within the hemolymph of laboratory-bred and experimentally infected ticks^60^ as their exposure to a higher inoculum concentration is less likely to affect the mass spectra acquisition. In this study we used DNA-based assays for the identification of infected specimens, as the limited number of pathogen-specific infected ticks would not allow a proper comparison of mass spectra profiles from infected and non-infected tick populations. Gene-based PCR was chosen as the pathogen screening method of this study. Although this approach could limit microorganism detection, we used several assays targeting most bacterial species previously described in *Dermacentor* spp.^6,55,63–72^. In addition, the robust number of specimens tested accounts for the influence of environmental traits, blood-meal of host species, and tick immunity on its microorganism diversity, similarly to the descriptions from 16S based-microbiota analysis or its microbiome^73^. As the main goal was to identify infected ticks to exclude from the reference database, future work might address how a robust reference database composed of infected samples may influence the MALDI-TOF ability to accurately identify pathogenic and non-pathogenic organisms harbored in field-collected ticks.

In summary, the data described herein is unique on several levels. Our use of MALDI-TOF MS is the first application of this technology to evaluate medically relevant tick species on a nationwide scale in the United States. This work also demonstrates the versatility, accuracy, and broad applicability of this technology to distinguish tick species that, until now, required the expertise of tick taxonomists to identify extremely subtle morphological differences, or relatively more expensive molecular methods to reveal genetic differences. Finally, our dataset represents the first creation of a publicly available tick protein database, now hosted on CDC MicrobeNet, to leverage the broad application of this platform for investigators worldwide. The establishment of this widely accessible and novel tool for the accurate identification of medically relevant tick species, corroborated by classical taxonomic and molecular methods, has important applications in public health research and surveillance. More rapid and cost-effective methods are needed to support national efforts that identify and monitor medically relevant ticks and their associated pathogens^7^ and MALDI-TOF MS could be added as a valuable tool to provide accurate and predictive data for tick and tickborne pathogen surveillance.

## Conclusion

In this investigation, we adopted MALDI-TOF MS technology as an integrative taxonomic tool to support the recent separation of *Dermacentor similis* from *D. variabilis* using morphological and high-throughput DNA-based identification methods. By creating a well-validated and robust protein-based dataset, accompanied by ample morphological and genetic information, we provide a proof-of-concept for this highly specific technique that leverages the speciation and subpopulation tick identification, particularly for species that pose challenges to accurate identification.

## Materials and methods

### Tick collection

A total of 492 adult *Dermacentor* spp. ticks were evaluated in this study, representing 54 sites within 19 western, eastern, and midwestern states from across the US (Fig. 1). Live specimens were collected between 2019 and 2022 by dragging/flagging methods, stored individually in 70–95% ethanol at room temperature (22–25 °C) until morphological, molecular DNA-based and MALDI-TOF MS analyses were performed. In addition to the contemporary specimens, 207 *Dermacentor* specimens from the United States National Tick Collection (from the U.S. National Museum of Natural History, Smithsonian Institution, housed at Georgia Southern University) were selected for a retrospective morphological analysis. The group represented four western US states; Washington (n = 94), Idaho (n = 36), Oregon (n = 31), and California (n = 46), collected between 1910 and 1986, preserved in absolute ethanol from the time of collection.

### Morphological identification

Tick speciation was determined initially by morphological identification performed under a stereo microscope at a magnification of 40x (Zeiss Stemi 305), according to previously established taxonomic identification keys^43,44,61,62^. For *D. similis*, specimens were additionally compared to descriptions from Lado and collaborators^32^. For record purposes, some specimens were selected for photographs of the dorsal and ventral sides, including images of the spiracular plate.

### DNA extraction and pathogen screening

After morphological identification, ticks were individually dissected for protein and DNA-based assays using 4 legs and body, respectively. Extraction of genomic DNA (gDNA) was performed individually using the DNeasy® Blood & Tissue Kit (Qiagen) following the manufacturer’s instructions. Extracted gDNA was stored at − 20 °C until needed for *Rickettsia*, *Anaplasma*, *Ehrlichia*, *Neoehrlichia*, *Neorickettsia*, *Wolbachia*, and *Anaplasma bovis*-like real-time PCR assays targeting 23S rDNA, *omp*A, 16S rDNA or *glt*A, *rrs*, and *gro*EL genes, respectively, as previously described^6,55,63–72^ (Table S1). PCR positive samples were further sequenced to identify the microorganism to species level.

### Tick sequencing and phylogenetic analysis

DNA from 105 morphologically identified *Dermacentor* spp. ticks (from 12 states) were analyzed for genetic characterization. A 365 bp fragment of the nuclear DNA internal transcribed spacer 2 (ITS2)^72^, and a 335 bp mtDNA fragment from the 12S ribosomal DNA gene sequence^55^ were both amplified. Purified PCR products were bidirectionally sequenced on an Applied Biosystems 3500 genetic analyzer using a BigDye Terminator V3.1 kit (Applied Biosystems). Amplicons were assembled and analyzed using Geneious Prime® 2022.0.2, and unique sequences were deposited in GenBank (https://www.ncbi.nlm.nih.gov/genbank/) (Table S2). Unique sequences were aligned in Geneious, and phylogenetic trees were inferred by the Bayesian method. Bayesian analyses were performed using the MrBayes 3.2.6 program within Geneious Prime. The general time reversible (GTR) model was utilized as the substitution model and *Dermacentor occidentalis* was used as an outgroup. A gamma model of variable rates across sites was used, and 1,100,000 generations were employed with four range categories. Support values at branch nodes were calculated as posterior probabilities by MrBayes; nodes with less than 0.90 posterior probability were collapsed to polytomies.

### Sample preparation for MS analysis

After the morphological identification was performed, all specimens were dissected with sterile scalpels under a stereo microscope at a magnification of 20x (Zeiss Stemi 305). For protein extraction to MALDI-TOF MS analysis, four legs per tick specimen were dissected and transferred to a separate tube, to air dry within a biosafety cabinet for 2 h. A previous study demonstrated that analysis of three legs provides sufficient quantity of tick proteins to generate quality spectra for intra-species reproducibility and inter-species differentiation^42^. After that, 50 µL of 100% acetonitrile was added and tubes were centrifuged for 2 min at 13,000 xg at room temperature. The supernatant was decanted, and any residual acetonitrile was removed using a micro pipettor. Specimens were homogenized in 20 µL of each of 70% formic acid and 100% acetonitrile (v/v) using a 5 mm stainless steel bead (Qiagen) on a TissueLyser II (Qiagen) for 90 s at 30 Hz. Tubes were then centrifuged at 13,000×*g* for 2 min. One µL of the final supernatant was deposited onto a spot of an MSP 96 target polished steel BC plate (Bruker) in 8 replicates for reference database construction runs or 3 replicates for validation runs. A bacterial test standard (Bruker) was also loaded in 8 replicates onto each plate to serve as a control. After drying, 1 μL of matrix solution composed of alpha-cyano-4-hydroxycinnamic acid HCCA (Bruker) was overlaid on each spot and left to dry for 10 min at room temperature. The plate was then transferred to a MALDI-TOF MS microflex® LRF instrument (Bruker).

### MALDI-TOF MS tick reference database and validation

As previously cited, a MALDI-TOF MS microflex® LRF system with the flexControl™ software (Bruker) was used to generate the protein spectra from each specimen. Spectra were acquired in the range of 2,000 to 20,000 m/z on a linear positive ion mode, using the company’s automated spectral quality engine AutoXecute™ within flexControl™. All operations, including control of spot position, laser power applied, and the number of shots collected also followed the automated AutoXecute™ engine.

For the reference database construction, a total of 24 single mass spectra of the 8 technical replicates were collected from each sample. Compass Explorer 4.1 was used for database management. Within this tool, each single spectrum was preprocessed to assess the mass spectra quality, number and frequency of ion peaks, and their intensity. A software-based algorithm for baseline subtraction and smoothing was applied at this step of the analysis. For quality control purposes, the calculation of main spectra profile (MSP) projections was based on a minimum of 20 single spectra compiled with a peak frequency cutoff of 70% between technical replicates. Samples that did not meet these criteria were considered low quality and consequently discarded. Only the high quality and reproducible MSPs were stored for the construction of the reference database, which are available at the CDC MicrobeNet Tick Module website (MicrobeNet). In addition, the same set of spectra used as a reference database were also exported to BioNumerics 7.6 (bioMérieux) for downstream analysis, including clustering.

For database validation purposes, 90 samples were processed to protein level using the previously described extraction procedure and run in flexControl™ using AutoXecute™. Two technical replicates were accessed from each sample with a single mass spectrum collected from each replicate. Each spectrum was then analyzed by comparison of log score values (LSV) of MS queries against the existing reference database using the MALDI-Biotyper MBT Compass v3.0. software (Bruker Daltonics). LSV reflects the confidence degree of the match between the queried MS spectra and those available in the MS reference spectra database. This score value supports the accuracy of specimen identification after comparison to a threshold value that can vary between 0 and 3. In accordance with previous arthropod-based studies, an LSV threshold of ≥ 1.7 indicates high confidence species level identification for arthropod analysis purposes^33,51^.

### Supplementary Information

Supplementary Figures.

Supplementary Tables.

## Data Availability

All tick MALDI-TOF spectra data is available for search at MicrobeNet. The authors also welcome requests to access the data used for the reference database construction upon reasonable request. All ITS2 nuclear DNA and 12S ribosomal DNA tick variants analyzed in this report are included in the manuscript within its correspondent NCBI accession numbers.
