# Supplementary material for: Applying MALDI-TOF MS to resolve morphologic and genetic similarities between two Dermacentor tick species of public health importance
Source: Sci Rep. 2024 Aug 27;14:19834. doi: 10.1038/s41598-024-69768-8 (PMC11350171; doi:10.1038/s41598-024-69768-8)
Supplement: Supplementary file 1 — Supplementary Figures. [file 41598_2024_69768_MOESM1_ESM.pdf]

APPLYING MALDI-TOF MS TO RESOLVE MORPHOLOGIC AND GENETIC SIMILARITIES BETWEEN TWO *DERMACENTOR* TICK SPECIES OF PUBLIC HEALTH IMPORTANCE

Maria F. B. M. Galletti[1*], Joy A. Hecht[1], John R. McQuiston[2], Jarrett Gartin[2], Jake Cochran[2], Bessie H. Blocher[1], Bryan N. Ayres[1], Michelle E. J. Allerdice[1], Lorenza Beati[3], William L. Nicholson[1], Alyssa N. Snellgrove[1], Christopher D. Paddock[1], US Tick MALDI-TOF consortium

[1] Rickettsial Zoonoses Branch, Division of Vector-Borne Diseases, National Center for Emerging and Zoonotic Infectious Diseases, Centers for Disease Control and Prevention, Atlanta, USA.

[2] Division of High-Consequence Pathogens and Pathology, National Center for Emerging and Zoonotic Infectious Diseases, Centers for Disease Control and Prevention, Atlanta, USA.

[3] United States National Tick Collection, Institute for Coastal Plain Science, Georgia Southern University, Statesboro, USA.

[*]Address correspondence to Maria F. B. M. Galletti, myu8@cdc.gov

Supplementary Information
Figures S1 and S2

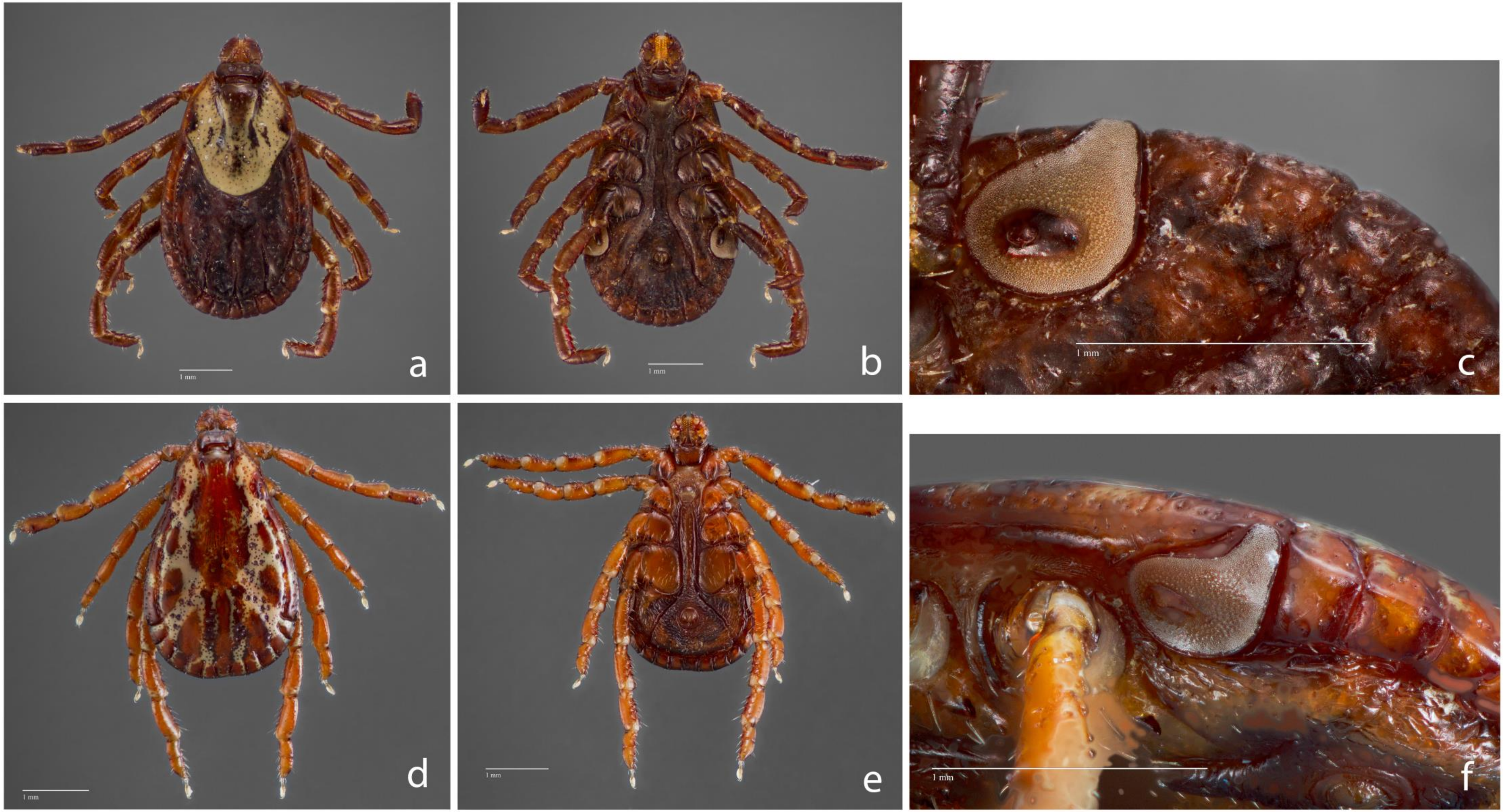

Figure S1. Historical *Dermacentor similis* samples part of the United States National Tick Collection, from the U.S. National Museum of Natural History, Smithsonian Institution, housed at Georgia Southern University. Washington *D. similis* female dorsal (**a**) and ventral (**b**) views, collected in 1969; Oregon *D. similis* male dorsal (**d**) and ventral (**e**) collected in 1974. Spiracular plates detailed from the respective specimen on the left (**c** and **f**).

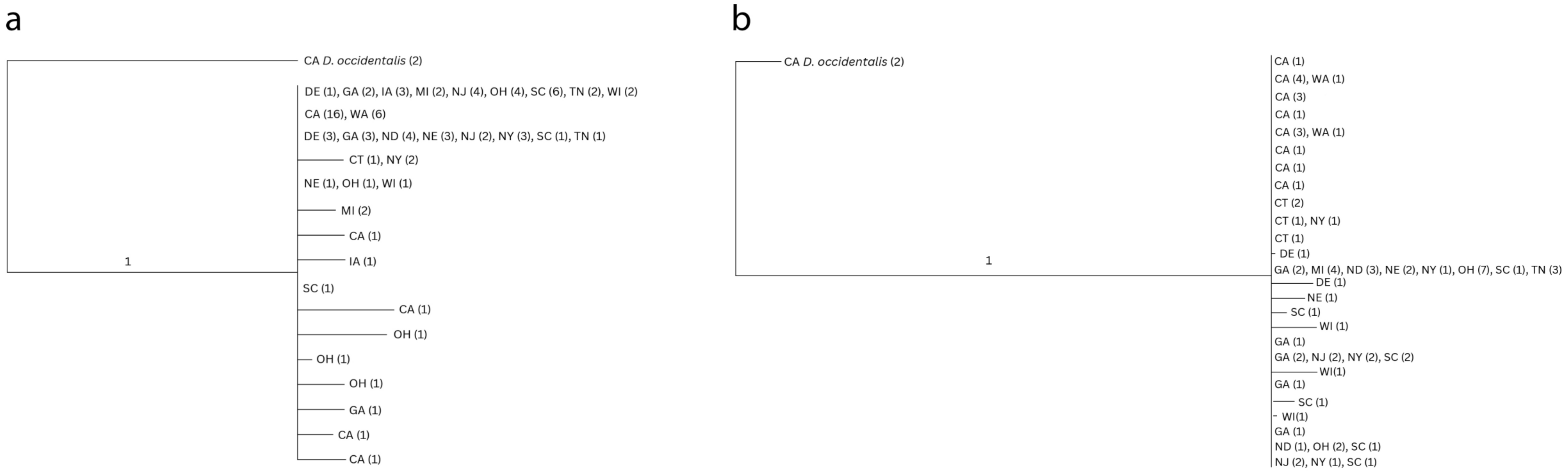

Figure S2. *Dermacentor* Bayesian analysis (BA) phylogenetic trees. (**a**) ITS2 nuclear DNA (344 base pairs) phylogenetic tree of 86 specimens and (**b**) 12S ribosomal DNA (287 base pairs) phylogenetic tree of 72 specimens. Larger clades include the group of specimens from the western US states (California and Washington) and the group of specimens from eastern and midwestern US regions. Numbers on nodes represent posterior probabilities. Numbers on parenthesis represent the number of identical sequences within that particular US state. *Dermacentor occidentalis* (Do) samples from California were used as an outgroup.
