# Supplementary material for: Applying MALDI-TOF MS to resolve morphologic and genetic similarities between two Dermacentor tick species of public health importance
Source: Sci Rep. 2024 Aug 27;14:19834. doi: 10.1038/s41598-024-69768-8 (PMC11350171; doi:10.1038/s41598-024-69768-8)
Supplement: Supplementary file 2 — Supplementary Tables. [file 41598_2024_69768_MOESM2_ESM.pdf]

Manuscript ID: facd1a0d-55ac-482f-9c20-30bf8ce038aa v2.0

Supplementary Information

Tables S1 and S2

APPLYING MALDI-TOF MS TO RESOLVE MORPHOLOGIC AND GENETIC SIMILARITIES BETWEEN TWO *DERMACENTOR* TICK SPECIES OF PUBLIC HEALTH IMPORTANCE

Maria F. B. M. Galletti[1*], Joy A. Hecht[1], John R. McQuiston[2], Jarrett Gartin[2], Jake Cochran[2], Bessie H. Blocher[1], Bryan N. Ayres[1], Michelle E. J. Allerdice[1], Lorenza Beati[3], William L. Nicholson[1], Alyssa N. Snellgrove[1], Christopher D. Paddock[1], US Tick MALDI-TOF consortium

[1] Rickettsial Zoonoses Branch, Division of Vector-Borne Diseases, National Center for Emerging and Zoonotic Infectious Diseases, Centers for Disease Control and Prevention, Atlanta, USA.

[2] Division of High-Consequence Pathogens and Pathology, National Center for Emerging and Zoonotic Infectious Diseases, Centers for Disease Control and Prevention, Atlanta, USA.

[3] United States National Tick Collection, Institute for Coastal Plain Science, Georgia Southern University, Statesboro, USA.

[*]Address correspondence to Maria F. B. M. Galletti, myu8@cdc.gov

Table S1. Oligonucleotide used in the pathogen screening and tick gene sequencing analyzes.

TARGET ORGANISM	GENE TARGET	OLIGONUCLEOTIDE NAME	SEQUENCE 5'-3'	PCR TYPE	AMPLICON SIZE (bp)	PRIMER DESIGN REFERENCES
Tick	12S rDNA	T1B	AAACTAGGATTAGATACCCT	PCR	335	55
		T2A	AATGAGAGCGACGGGCGATGT			
Tick	ITS2*	DITS2_F1	GTTTGCGAGAGAGTCGGAAG	PCR	365	72
		DITS2_R1	CTGGAGTGGCCCAGTCTTT			
Genus *Rickettsia*	23S rRNA (rrl)	PanR8_F	AGCTTGCTTTTGGATCATTTGG	real-time PCR	111	68
		PanR8_R	TTCCTTGCCTTTTCATACATCTAGT			
		PanR8_P	CCTGCTTCTATTTGTCTTGCAGTAACACGCCA			
Rickettsia akari, *R. amblyommatis*, *R. australis*, *R. canadensis*, *R. conorii*, *R. felis*, *R. honei*, *R. massiliae*, *R. montanensis*, *R. parkeri*, *R. rhipicephali*, *R. sibirica*, *R. slovaca*, *R. rickettsii*, *R. prowazekii*, and *R. typhi*	*omp*A*	RR190-70	ATGGCGAATATTTCTCCAAAA	Primary stage Nested PCR	632	63,64
		RR190-701	GTTCCGTTAATFGCAGCATCT			
		RR190-70	ATGGCGAATATTTCTCCAAAA	Semi-nested stage PCR	532	
		RR190-602	AGTGCAGCATTCGCTCCCCCT			
Genera *Anaplasma*, *Ehrlichia*, *Neoehrlichia*, *Neorickettsia*, and *Wolbachia*	16S	ECHSYBR-F	AACACATGCAAGTCGAACGG	real-time PCR	146 to 154	67
		ECHSYBR-R	CCCCCGCAGGGATTATACA			
Rickettsia bellii	*glt*A*	RBELLIF	ATCCTGATTTGCTGAATTTTTT	real-time PCR	338	69
		RBELLIR	TGCAATACCAGTACTGACG			
		RBELLII_P3_CR610	ATGATGTTTGCCACACCTTGTGAAAA			
Anaplasma bovis-like	rrs	Out2F	GATAGCGGAATTCCTAGTGTAGAGGTG	PCR	599	6,71
		317Pan	AAAGGAGGTAATCCAGC			
	*glt*A*	Abov_gltA2F	CGGAAATTACTTTTATAGATGG	PCR	826	6
		Abov_gltA2R	CATACCAYTGAGAAACCCAAC			
	*gro*EL	HS1-f	CGTCAGTGGGCTGGTAATGAA	Primary stage Nested PCR	1360	65, 70
		HS6-r	CCWCCWGGTCWACACCTTC			
		HS3-f	ATAGTYATGAAGGAGAGTGAT	Semi-nested stage PCR	1320	70
		HSV-r	TCAACAGCAGCTCTAGTWG			

*ITS2: internal transcribed spacer 2 region; *omp*A: outer membrane protein A; *glt*A: citrate synthase.

Table S2. Accession numbers of the nuclear ITS2 and mitochondrial 12S ribosomal sequences deposited in NCBI.

Target	Organism	Accession number	Sample	Geographical origin (USA state)
internal transcribed spacer 2	*Dermacentor variabilis*	OR757077	*Dermacentor variabilis* TN 114	TN
internal transcribed spacer 2	*Dermacentor variabilis*	OR757078	*Dermacentor variabilis* OH 731	OH
internal transcribed spacer 2	*Dermacentor variabilis*	OR757079	*Dermacentor variabilis* DE 2383	DE
internal transcribed spacer 2	*Dermacentor variabilis*	OR757080	*Dermacentor variabilis* GA 2606	GA
internal transcribed spacer 2	*Dermacentor variabilis*	OR757081	*Dermacentor variabilis NJ 2292*	NJ
internal transcribed spacer 2	*Dermacentor variabilis*	OR757082	*Dermacentor variabilis IA 3267*	IA
internal transcribed spacer 2	*Dermacentor variabilis*	OR757083	*Dermacentor variabilis SC 940*	SC
internal transcribed spacer 2	*Dermacentor variabilis*	OR757084	*Dermacentor variabilis WI 1532*	WI
internal transcribed spacer 2	*Dermacentor variabilis*	OR757085	*Dermacentor variabilis MI 2368*	MI
internal transcribed spacer 2	*Dermacentor variabilis*	OR757086	*Dermacentor variabilis ND 1353*	ND
internal transcribed spacer 2	*Dermacentor variabilis*	OR757087	*Dermacentor variabilis DE 2399*	DE
internal transcribed spacer 2	*Dermacentor variabilis*	OR757088	*Dermacentor variabilis NE 3145*	NE
internal transcribed spacer 2	*Dermacentor variabilis*	OR757089	*Dermacentor variabilis GA 2601*	GA
internal transcribed spacer 2	*Dermacentor variabilis*	OR757090	*Dermacentor variabilis NY 1721*	NY
internal transcribed spacer 2	*Dermacentor variabilis*	OR757091	*Dermacentor variabilis NJ 2291*	NJ
internal transcribed spacer 2	*Dermacentor variabilis*	OR757092	*Dermacentor variabilis TN 115*	TN
internal transcribed spacer 2	*Dermacentor variabilis*	OR757093	*Dermacentor variabilis NY 1719*	NY
internal transcribed spacer 2	*Dermacentor variabilis*	OR757094	*Dermacentor variabilis CT 2063*	CT
internal transcribed spacer 2	*Dermacentor variabilis*	OR757095	*Dermacentor variabilis OH 729*	OH
internal transcribed spacer 2	*Dermacentor variabilis*	OR757096	*Dermacentor variabilis WI 1531*	WI
internal transcribed spacer 2	*Dermacentor variabilis*	OR757097	*Dermacentor variabilis IA 3280*	IA
internal transcribed spacer 2	*Dermacentor variabilis*	OR757098	*Dermacentor variabilis OH 741*	OH
internal transcribed spacer 2	*Dermacentor variabilis*	OR757099	*Dermacentor variabilis NE 3136*	NE
internal transcribed spacer 2	*Dermacentor variabilis*	OR757100	*Dermacentor variabilis GA 2604*	GA
internal transcribed spacer 2	*Dermacentor variabilis*	OR757101	*Dermacentor variabilis MI 2369*	MI
internal transcribed spacer 2	*Dermacentor variabilis*	OR757102	*Dermacentor variabilis OH 727*	OH
internal transcribed spacer 2	*Dermacentor variabilis*	OR757103	*Dermacentor variabilis OH 724*	OH
internal transcribed spacer 2	*Dermacentor variabilis*	OR757104	*Dermacentor variabilis SC 924*	SC
small subunit ribosomal RNA gene	*Dermacentor variabilis*	OR840660	*Dermacentor variabilis OH 727*	OH
small subunit ribosomal RNA gene	*Dermacentor variabilis*	OR840661	*Dermacentor variabilis ND 1353*	ND
small subunit ribosomal RNA gene	*Dermacentor variabilis*	OR840662	*Dermacentor variabilis WI 1530*	WI
small subunit ribosomal RNA gene	*Dermacentor variabilis*	OR840663	*Dermacentor variabilis MI 2371*	MI
small subunit ribosomal RNA gene	*Dermacentor variabilis*	OR840664	*Dermacentor variabilis GA 2606*	GA
small subunit ribosomal RNA gene	*Dermacentor variabilis*	OR840665	*Dermacentor variabilis TN 114*	TN
small subunit ribosomal RNA gene	*Dermacentor variabilis*	OR840666	*Dermacentor variabilis NY 1719*	NY
small subunit ribosomal RNA gene	*Dermacentor variabilis*	OR840667	*Dermacentor variabilis NE 3145*	NE
small subunit ribosomal RNA gene	*Dermacentor variabilis*	OR840668	*Dermacentor variabilis SC 928*	SC
small subunit ribosomal RNA gene	*Dermacentor variabilis*	OR840669	*Dermacentor variabilis SC 924*	SC
small subunit ribosomal RNA gene	*Dermacentor variabilis*	OR840670	*Dermacentor variabilis ND 1356*	ND
small subunit ribosomal RNA gene	*Dermacentor variabilis*	OR840671	*Dermacentor variabilis OH 736*	OH

Table S2. continuation.

Target	Organism	Accession number	Sample	Geographical origin (USA state)
small subunit ribosomal RNA gene	*Dermacentor variabilis*	OR840672	*Dermacentor variabilis* WI 1529	WI
small subunit ribosomal RNA gene	*Dermacentor variabilis*	OR840673	*Dermacentor variabilis* NY 1721	NY
small subunit ribosomal RNA gene	*Dermacentor variabilis*	OR840674	*Dermacentor variabilis* NJ 2293	NJ
small subunit ribosomal RNA gene	*Dermacentor variabilis*	OR840675	*Dermacentor variabilis* GA 2601	GA
small subunit ribosomal RNA gene	*Dermacentor variabilis*	OR840676	*Dermacentor variabilis* SC 943	SC
small subunit ribosomal RNA gene	*Dermacentor variabilis*	OR840677	*Dermacentor variabilis* SC 938	SC
small subunit ribosomal RNA gene	*Dermacentor variabilis*	OR840678	*Dermacentor variabilis* SC 930	SC
small subunit ribosomal RNA gene	*Dermacentor variabilis*	OR840679	*Dermacentor variabilis* NY 1797	NY
small subunit ribosomal RNA gene	*Dermacentor variabilis*	OR840680	*Dermacentor variabilis* NJ 2291	NJ
small subunit ribosomal RNA gene	*Dermacentor variabilis*	OR840681	*Dermacentor variabilis* DE 2399	DE
small subunit ribosomal RNA gene	*Dermacentor variabilis*	OR840682	*Dermacentor variabilis* SC 940	SC
small subunit ribosomal RNA gene	*Dermacentor variabilis*	OR840683	*Dermacentor variabilis* CT 2063	CT
small subunit ribosomal RNA gene	*Dermacentor variabilis*	OR840684	*Dermacentor variabilis* CT 2065	CT
small subunit ribosomal RNA gene	*Dermacentor variabilis*	OR840685	*Dermacentor variabilis* NY 1723	NY
small subunit ribosomal RNA gene	*Dermacentor variabilis*	OR840686	*Dermacentor variabilis* CT 2130	CT
small subunit ribosomal RNA gene	*Dermacentor variabilis*	OR840687	*Dermacentor variabilis* SC 927	SC
small subunit ribosomal RNA gene	*Dermacentor variabilis*	OR840688	*Dermacentor variabilis* NE 3235	NE
small subunit ribosomal RNA gene	*Dermacentor variabilis*	OR840689	*Dermacentor variabilis* WI 1531	WI
small subunit ribosomal RNA gene	*Dermacentor variabilis*	OR840690	*Dermacentor variabilis* WI 1532	WI
small subunit ribosomal RNA gene	*Dermacentor variabilis*	OR840691	*Dermacentor variabilis* GA 2600	GA
small subunit ribosomal RNA gene	*Dermacentor variabilis*	OR840692	*Dermacentor variabilis* GA 2605	GA
small subunit ribosomal RNA gene	*Dermacentor variabilis*	OR840693	*Dermacentor variabilis* DE 2384	DE
internal transcribed spacer 2	*Dermacentor similis*	OR734979	*Dermacentor similis* CA 2666	CA
internal transcribed spacer 2	*Dermacentor similis*	OR734980	*Dermacentor similis* WA 3059	WA
internal transcribed spacer 2	*Dermacentor similis*	OR734981	*Dermacentor similis* CA 2680	CA
internal transcribed spacer 2	*Dermacentor similis*	OR734982	*Dermacentor similis* CA 2761	CA
internal transcribed spacer 2	*Dermacentor similis*	OR734983	*Dermacentor similis* CA 2738	CA
internal transcribed spacer 2	*Dermacentor similis*	OR734984	*Dermacentor similis* CA 2686	CA
small subunit ribosomal RNA gene	*Dermacentor similis*	PP085218	*Dermacentor similis* CA 2664	CA
small subunit ribosomal RNA gene	*Dermacentor similis*	PP085219	*Dermacentor similis* WA 3065	WA
small subunit ribosomal RNA gene	*Dermacentor similis*	PP085220	*Dermacentor similis* CA 2683	CA
small subunit ribosomal RNA gene	*Dermacentor similis*	PP085221	*Dermacentor similis* WA 3092	WA
small subunit ribosomal RNA gene	*Dermacentor similis*	PP085222	*Dermacentor similis* CA 2686	CA
small subunit ribosomal RNA gene	*Dermacentor similis*	PP085223	*Dermacentor similis* CA 2658	CA
small subunit ribosomal RNA gene	*Dermacentor similis*	PP085224	*Dermacentor similis* CA 2680	CA
small subunit ribosomal RNA gene	*Dermacentor similis*	PP085225	*Dermacentor similis* CA 2663	CA
small subunit ribosomal RNA gene	*Dermacentor similis*	PP085226	*Dermacentor similis* CA 2736	CA
small subunit ribosomal RNA gene	*Dermacentor similis*	PP085227	*Dermacentor similis* CA 2738	CA
